# Degree of severity of molar incisor hypomineralization and its relation to dental caries

**DOI:** 10.1038/s41598-018-19821-0

**Published:** 2018-01-19

**Authors:** A. Negre-Barber, J. M. Montiel-Company, M. Catalá-Pizarro, J. M. Almerich-Silla

**Affiliations:** Departament of Stomatology, Faculty of Medicine and Dentistry, University of Valencia, Valencia, Spain

## Abstract

Molar incisor hypomineralization is a developmental defect of dental enamel associated with rapid caries progression. In order to discover whether molar incisor hypomineralization predisposes to dental caries, a cross-sectional cohort study was conducted in a sample of 414 children aged between eight and nine years. It was found that 24.2% of the children presented molar incisor hypomineralization. Of these, 72% had a mild form and 28% a severe form. Caries prevalence was greater among the children with severe form (60.7%) than in those with mild form (43.1%) or no molar incisor hypomineralization (45.5%). The caries indices were higher in out molar incisor hypomineralization (1.18) or with mild form (1.08). The tooth-surface caries ratio was significantly higher in surfaces with severe hypomineralization than in those with no hypomineralization or mild hypomineralization. A linear regression model showed that cariogenic food intake and the presence of severe molar incisor hypomineralization were significantly associated with DMFS. Consequently, an association was found to exist between dental caries and the presence of surfaces affected by severe molar incisor hypomineralization, which should be considered a risk factor within the multifactorial etiology of caries.

## Introduction

Dental caries and developmental defects of enamel (DDE) are currently the most frequent problems observed in primary dentition and early permanent dentition. DDE are due to faulty enamel formation, which makes the enamel more susceptible to attack by acids and, therefore, to dental caries. The defective enamel provides an ideal environment for plaque adhesion and colonization by cariogenic bacteria, enabling lesions to progress rapidly^1,2^. DDE include molar incisor hypomineralization (MIH)^3^, which has prevalence rates ranging between 2.9% and 44% in different countries^4^.

Molar incisor hypomineralization (MIH) is a mineralization disorder that affects the permanent first molars and, on occasion, the permanent incisors. It can present in a mild form consisting of opacities with a white/yellow/brown tone of color, or a severe form with post-eruptive enamel breakdown (PEB), caries, atypical restorations and extractions^3^. Yellow and brown opacities are more porous than white ones, have worse prism organization and are easier for acids to attack^5^. In the most severe cases, the cusps and occlusal surfaces of the molars can disintegrate, favoring the appearance of a rapid progression of caries that does not form part of the childhood caries pattern^6^. Owing to the greater porosity of the enamel and its lower mechanical resistance^7^, MIH is considered a risk factor for dental caries in populations with low caries levels^8–14^.

In areas with high caries prevalence, MIH can remain undetected because the rapid advance of the caries eliminates any trace of the hypomineralization^15^. In areas where caries prevalence is moderate to low, however, the two conditions can coexist and can be differentiated as long as the MIH is not severe. If caries occurs, the mineralization disorder favors its rapid progress, causing major crown destruction and loss of the tooth. Additionally, the tooth sensitivity experienced by most of the children affected by MIH reduces oral hygiene and self-cleaning, increasing the risk of caries.

Most of the authors who have studied the association between MIH and dental caries^8,10,11,15–22^ have shown a relationship between increased DMFT and children with MIH compared to those without MIH. However, others such as Dietrich *et al*.^23^, Calderara *et al*.^24^ or Heitmüller *et al*.^25^ have not found a significant association between dental caries and MIH.

Appropriate exposure to fluoride and the introduction of programs to prevent and control etiological factors have made a decisive contribution to reducing dental caries, even though it continues to be a prevalent disease in the child population. The rise in MIH prevalence makes it necessary to study its possible relationship with dental caries. Since MIH seems to increase the risk of caries mainly in the permanent first molars, the aim of the present study was to discover whether there is a relationship between dental caries and MIH, and its distribution in a sample of 8 to 9 year-old children.

## Materials and Methods

### Ethical considerations

The clinical study was authorized by the University of Valencia’s human research ethics committee, under procedure number H1372162226937, in accordance with the Helsinki Declaration on Medical Research Involving Human Subjects. The procedures were explained clearly to all the parents/guardians and participants before their inclusion in the study and informed consent to oral examination and intraoral photography was obtained from all the parents/guardians of the children participating. The methods employed were applied in accordance with approved guidelines.

### Study design

This cross-sectional study was part of a wider research project named INMA (the Spanish initials for infancy, childhood and the environment), which investigates the effects of environmental factors on the development and health of a cohort of children in the Valencia region of Spain whose mothers were recruited by consecutive sampling in early pregnancy and have been followed-up since their inclusion in 2003–2004^26,27^. These children are examined at regular intervals and the 8–9 year-old appointment provided the opportunity to perform an oral examination.

### Sample size calculation

A minimum sample size of 405 subjects was considered sufficient to estimate MIH prevalence with a 95% confidence level and a level of precision of +/−4% in a population with an MIH level of around 20%. The final sample comprised 414 children aged 8 and 9 years (born between 2004 and 2005) who attended the 8–9 year-old check-up, which included an oral examination.

### Diagnostic criteria

Prior to the calibration session, the dentist who was to carry out the MIH examinations and a dental practitioner with experience in diagnosing and treating MIH conducted a standardization session. The diagnostic criteria were established as being those drawn up by the EAPD in 2003^3^, which match the current basis for studies of MIH^4,28^. They are as follows: white/yellow/brown demarcated opacities, considered a mild degree of MIH; or post-eruptive enamel breakdown associated with opacities, extensive caries with surrounding opacities or in low-risk surfaces, atypical restorations of a size and location unrelated to the caries pattern, teeth with crowns if MIH is found in other teeth, or extractions due to MIH, which were considered a mild degree of MIH. The occlusal/incisal, labial and lingual/palatal surfaces of the permanent incisors and permanent first molars were assessed. Spots larger than 1 mm were classed as hypomineralized. MIH was diagnosed when a permanent first molar was affected by hypomineralization. Incisors were only diagnosed as MIH if a molar was affected. The degree of severity was decided by the most severe disorder in the child’s mouth.

For caries diagnosis, the ICDAS II criteria were employed^29,30^. ICDAS grades 1 to 6 range from white spots to extensive caries. In grade 1 a white spot or visual change in the enamel is visible on drying the tooth; in grade 2 the white spot is visible on the moist tooth; in grade 3, localized enamel breakdown (with no visible dentin) is observed; in grade 4 there is dentinal shadow (with no visible dentin); in grade 5, a cavity with visible dentine, and in grade 6, an extensive cavity with visible dentine affecting over half of a tooth surface.

The teeth examined were the index teeth for MIH (permanent first molars and permanent incisors). The DMFS index was calculated from the sum of the decayed (ICDAS codes 1 to 6), missing and filled surfaces of the teeth used to assess MIH.

### Calibration of the examiners

The first calibration session for MIH was conducted with 46 clinical photographs which were used to assess all the degrees of hypomineralization (no MIH, mild MIH and severe MIH), as well as other disorders involved in a differential diagnosis, such as fluorosis, hypoplasia and amelogenesis imperfecta. The diagnostic agreement was 100%. For dental caries, an online course in caries diagnosis using the ICDAS-II criteria^31^ was followed, which involved assessing images of different grades of dental caries.

A second calibration session was then carried out with 54 children who attended the pediatric dentistry unit in the stomatology department of the University of Valencia. The agreement measured by linear-weighted kappa was 0.83 for the MIH examiner and 0.91 for the dental caries examiner, which are considered good results on the Landis and Koch scale.

The data were collected by an MIH calibrated dentist and a different ICDAS II calibrated dentist and were recorded on dental charts to facilitate the data collection process and eliminate possible errors. A prior pilot study was conducted to clarify or correct any errors that arose.

### Examination

The examinations were carried out at the pediatric dentistry unit in the stomatology department of the University of Valencia. The equipment consisted of two dental chairs with lighting, cotton swabs for removing excess plaque or saliva, flat mouth mirrors and sterilized standard no. 4/6 double-ended exploration probes.

The teeth were first examined when wet and were then dried with cotton swabs for MIH diagnosis and with the equipment’s air jet, if necessary, for caries diagnosis. The data were collected on an examination record specifically prepared for this study, which had a section for personal details and a dental chart for recording the data on MIH and dental caries.

### Questionnaire

At the time of the oral examination, the children completed a questionnaire with 18 multiple-choice items concerning their health knowledge, oral hygiene habits and diet (data no shown). Based on the responses to this questionnaire, three categorized variables were generated: cariogenic food intake, teeth brushing habits and fluoride intake.

Cariogenic food intake was considered high if the child consumed sugary drinks or foods during and/or between main meals every day of the week, moderate if they were consumed between 4 and 6 days a week, and low if consumed a maximum of 3 days a week. Oral hygiene was considered correct when the child brushed his/her teeth at least once a day, poor if less than once a day and absent if the teeth were not brushed. Fluoride intake was considered good if the teeth were brushed with fluoride toothpaste more than once a day and a fluoride mouthwash was used weekly or every 2 weeks, moderate if the teeth were brushed with fluoride toothpaste only once a day and fluoride mouthwash was used occasionally, poor when brushing with fluoride toothpaste was performed less than once a day and no fluoride mouthwash was used, and absent when the teeth were brushed without fluoride toothpaste and no fluoride mouthwash was used.

### Statistical analysis

The completed examination records were entered into an Access® data base (Access 2003; Microsoft Corporation, Redmont, WA, USA) and transferred to an Excel® spreadsheet (Excel 2003; Microsoft Corporation, Redmont, WA, USA) for treatment by the SPSS Statistics 22.0® program (IBM SPSS, Chicago, IL, USA).

The study variables were: MIH diagnosis, MIH level (mild or severe), DMFS index and its components, caries prevalence (DMFS >0), tooth-surface caries ratio (the number of decayed or filled surfaces divided by the total number of exposed surfaces), cariogenic food intake (classed as low, moderate or high), oral hygiene habits (classed as correct, poor or absent) and fluoride intake (good, moderate, poor or absent).

Descriptive statistics were calculated, with means and confidence intervals for the quantitative variables and percentages and confidence intervals for the qualitative variables. The normality of the distributions was tested with Kolmogorov-Smirnov´s test. Student’s t-test or ANOVA were used to determine significant differences between means and a chi-squared test was used to detect differences in proportions. Taking the DMFS index as the dependent variable and cariogenic food intake, teeth brushing habits and fluoride intake as the independent variables, mild MIH and severe MIH were analyzed with a linear regression model. The level of significance was set at p < 0.05.

## Results

A total of 414 children (212 boys and 202 girls) with an average age of 9.16 years (95% CI 9.13–9.18) were examined. In total, 24.2% (100 children) had MIH. Of these, 72% had a mild form and 28% a severe form. No statistically significant differences by gender were encountered in a Chi squared test (p = 0.521). Within the group of children diagnosed as having MIH, the mean number of teeth affected were 2.7 permanent first molars and 1.3 permanent incisors.

The caries prevalence (DMFS >0) in the sample was 45.9%. In the children with MIH it was 48% and in those without MIH 45.5%, with no statistically significant differences (Chi^2^ p = 0.627). On comparing the children with MIH according to the degree of severity, however, significant differences in caries prevalence (Chi^2^ p = 0.0251) were encountered between those with mild MIH (43.1%) and with severe MIH (60.7%).

The DMFS index score for the total sample was 1.23. It was 1.40 for the children with MIH and 1.18 for those without MIH (Table 1). No statistically significant differences in DMFS score were encountered between these two groups (p = 0.30).

**Table 1:** DMFS distribution for children with and without MIH. Mean values with 95% CI in parentheses. For both groups, M was a constant equal to zero.

Mean values	**Total (n = 414)**	**No MIH (n = 314)**	**MIH (n = 100)**	**Mild MIH (n = 72)**	**Severe MIH (n = 28)**
Component D	1.11 (0.95–1.28)	1.08 (0.90–1.27)	1.22 (0.84–1.60)	1.03 (0.61–1.44)	1.71 (0.83–2.59)
Component F	0.11 (0.06–0.16)	0.09 (0.04–0.14)	0.18 (0.06–0.30)	0.06 (0.00–0.12)	0.50 (0.10–0.90)
DMFS index	1.23 (1.05–1.41)	1.18 (0.98–1.38)	1.40 (0.99–1.81)	1.08 (0.67–1.50)	2.21^1,2^ (1.23–3.20)

^1^Significant difference compared to no MIH according to post-hoc ANOVA (p < 0.05).

^2^Significant difference compared to mild MIH according to post-hoc ANOVA (p < 0.05).

The DMFS index score was 1.08 for the children with mild MIH and 2.21 for those with severe MIH. An ANOVA showed differences in DMFS (p = 0.012) (Table 1). The post hoc ANOVA indicated that the differences in DMFS were mostly between the children with no MIH and those with severe MIH (p = 0.013) and between those with mild MIH and severe MIH (p = 0.016). A linear tendency was observed, as the mean DMFS values increased in line with the degree of severity.

The tooth-surface caries ratio of the children without MIH was 0.04 (95% CI 0.03–0.05). For those with mild MIH it was 0.04 (95% CI 0.02–0.06) overall, but 0.02 (95% CI 0.01–0.03) in surfaces without hypomineralization and 0.11 (95% CI 0.06–0.15) where hypomineralization was present and the difference was statistically significant (p = 0.001). For children with severe MIH the ratio was 0.08 (95% CI 0.04–0.12), with 0.02 (95% CI 0.01–0.04) for surfaces without MIH, 0.12 (95% CI 0.02–0.20) for surfaces with mild hypomineralization and 0.40 (95% CI 0.22–0.59) for surfaces with severe hypomineralization.

**Figure 1:**
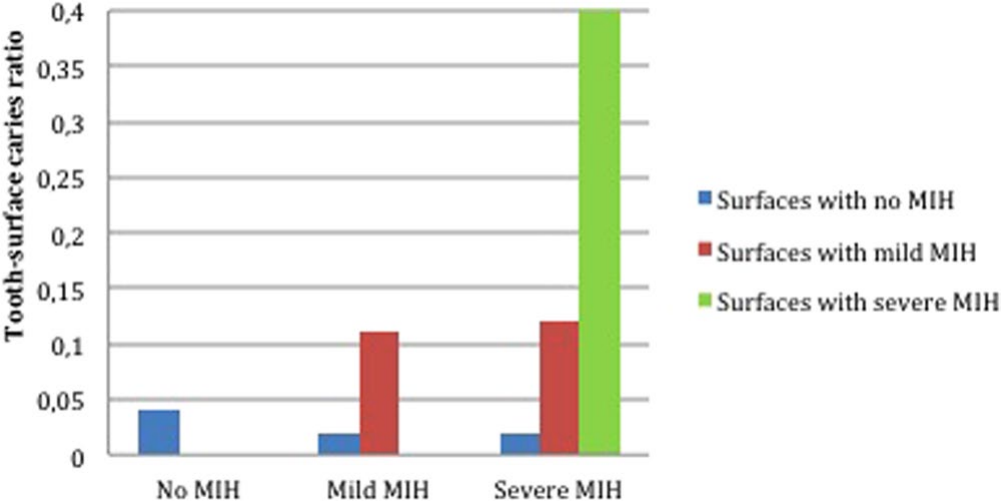
Tooth-surface caries ratio by hypomineralization level for each MIH group.

In the children with mild MIH, the tooth-surface caries ratio was greater for hypomineralized surfaces than for those with no hypomineralization, and the difference was statistically significant (p = 0.001). In the children with severe MIH, statistically significant differences in ratio were found between surfaces with mild hypomineralization and no hypomineralization (p = 0.038), and between surfaces with mild and severe hypomineralization (p = 0.01). There were no statistically significant differences in ratio between the three groups for surfaces without hypomineralization (p = 0.976), or between children with mild MIH and severe MIH for surfaces affected by mild hypomineralization (p = 0.871). The ratios are shown in Fig. 1.

The distribution of the cariogenic food intake, oral hygiene habits and fluoride intake variables in relation to the DMFS index is shown in Table 2. The only significant differences in DMFS were between low and high intake of cariogenic foods (post-hoc p = 0.012). Moreover, a linear trend was observed in the mean DMFS score, which rose as the cariogenic food intake increased (p = 0.012).

**Table 2:** Frequency of variables and mean DMFS score by variable.

		n	Frequency	Mean DMFS score	95% CI	ANOVA p-value
Cariogenic food intake	Low	229	55.3%	1.03	0.82–1.24	0.019*
	Moderate	147	35.5%	1.38	1.05–1.72	
	High	38	9.2%	1.84	1.20–2.47	
Oral hygiene habits	Correct	82	19.8%	1.15	0.74–1.56	0.108
	Poor	223	53.9%	1.10	0.88–1.33	
	Absent	109	26.3%	1.55	1.16–1.94	
Fluoride intake	Good	67	16.2%	1.22	0.78–1.66	0.700
	Moderate	173	41.8%	1.32	1.03–1.61	
	Poor	147	35.5%	1.19	0.91–1.48	
	Absent	27	6.5%	0.88	0.25–1.52	

*Significant difference (p < 0.05).

A linear regression model was constructed with the DMFS score as the dependent variable and cariogenic foods, tooth brushing, fluoride intake, mild MIH and severe MIH as the independent variables. The model obtained R^2^ = 0.044 and p = 0.002. Severe MIH (p = 0.002) and cariogenic food intake (p = 0.020) were shown to be significant and independent variables in this model (Table 3).

**Table 3:** Linear regression model fitting DMFS to the independent variables.

**Variables related to DMFS**	**β coefficients (unstandardized)**	**P value**
Constant	0.806	0.001
Cariogenic food intake	0.073	0.02*
Unfavorable tooth brushing habits	0.206	0.122
Fluoride intake	−0.089	0.411
Presence of mild MIH	−0.131	0.578
Presence of severe MIH	1.095	0.002*

*p < 0.05.

The final model constructed with the two significant variables met the linearity criterion (ANOVA p = 0.000). The residuals fulfilled the conditions of independence (Durbin-Watson = 1,94), homoscedasticity (absence of association in the dispersion plot between predicted values and residuals) and normality (Kolmogorv-Smirnov’s test p = 0.200). Finally there was no co-linearity between the two significant variables (Pearson correlation = −0.040).

## Discussion

MIH prevalence varies between 2.9% and 44% in different countries^4^. In Spain it ranges between 12.4% and 21.8%^8,32,33^, with 24.2% observed in the present study. MIH is most often encountered in a mild form^8,10,12,15,24,33–37^, which agrees with the results of the present study, where 72% of the MIH cases were mild and 28% severe.

Most authors have concluded that there is a relationship between hypomineralized teeth and dental caries^8,10,11,15–22^. However, others have stated that hypomineralization does not predispose towards caries in hypomineralized teeth^23–25^. In the present study, no statistically significant differences in caries prevalence were found between children with MIH (irrespective of level) and without MIH (48% and 45.5% respectively). However, on distinguishing between different levels of MIH, the severe MIH cases had significantly higher caries prevalence than those with mild MIH (60.7% and 43.1% respectively, p = 0.0251), which would indicate that more severe MIH entails a higher susceptibility to caries.

In the present study, as in Heitmuller *et al*.^25^, no statistically significant differences in DMFS between children with no MIH and those with mild MIH were observed. Few previous studies have distinguished between different levels of MIH when assessing differences in caries scores. In the present case, the caries scores were significantly higher in children with severe MIH compared to those with mild MIH or no MIH. Also, a linear tendency was found in mean DMFS scores, which rose as the severity of the hypomineralization increased.

It has been said that one of the main risks of observational bias when studying the relationship between MIH and caries is that the examiner who records the presence or absence of caries is necessarily also seeing the presence or absence of MIH, and vice versa, hence the recommendation that MIH and caries be assessed by two different examiners^38^. In this study, the MIH and caries evaluations, the latter using the ICDAS criteria, were conducted independently by two separate observers after calibration in the respective methods.

Several studies have shown that children with MIH need more dental treatment (whether urgent, non-urgent or preventive) than those without MIH^8,11,15,16,18,39^. MIH has an impact on increased caries prevalence and on restoration work in permanent first molars^16,18^. The higher scores for DMFS and its F component obtained in the present study confirm that children with severe MIH need more fillings.

The tooth-surface caries ratio rises as the severity of hypomineralization increases^5^. This could be because opacities in the cream to brown color range are more porous^5^ and more susceptible to PEB, and PEB, in turn, exacerbates the caries and increases its severity. Kosma *et al*.^22^ observed that the more severe the MIH the greater the caries, which agrees with Pitiphat *et al*.^20^, who found that caries lesions are 10 times more frequent in teeth with PEB (severe MIH) than in teeth that only have opacities (mild MIH). Elfrink *et al*.^40^ observed that the mean density of the hydroxyapatite in opacities in the yellow to brown color range is 20% to 22% lower than in sound enamel, while the difference is almost nonexistent in white opacities. The results of the present study have also shown that the caries is far greater in surfaces with severe MIH than in surfaces with mild MIH or no MIH.

The variability in the results of studies that have investigated the relationship between MIH and dental caries suggests the need for a standard protocol that would allow comparative analysis and strengthen the evidence for the conclusions. The present study followed the MIH diagnostic criteria established by EAPD^3^, which agree with the current guidelines proposed at the 12th EAPD Congress held in Sopot (Poland) in 2014 and published by Elfrink *et al*.^4^ and Ghanim *et al*.^28^.

This study provides detailed information on the caries status of teeth with MIH, distinguishing between caries in surfaces with mild hypomineralization or with severe hypomineralization in order to measure which level of the disease has a greater association with MIH. Caries was measured in the occlusal, labial and palatal/lingual surfaces of molars and incisors, the teeth that are liable to MIH. Other authors have also examined for caries in interproximal surfaces^8,10,35,41^ but these cannot be examined for MIH, which could lead to underestimating the relationship between MIH and caries. Also, in the present authors’ opinion, studies that include all the teeth^8,17,19–21,25,42–46^ can skew the results because of the greater number of teeth and because they cannot be compared with the index teeth for MIH.

As regards caries diagnosis, the criteria used in the different studies have also differed: most have followed the WHO criteria^8,15–17,19–22,24,39,42^ and very few have used ICDAS^18^ or the Universal Visual Scoring System^25^. The lack of agreement between the ICDAS^29,30^ and WHO^47^ diagnostic criteria could make it difficult to achieve an adequate assessment of the epidemiological trend in dental caries, which is why the EAPD prescribed the use of ICDAS II for caries prevalence studies^48^. The ICDAS II system enables initial stages of caries to be coded, unlike the WHO system, which only scores active caries lesions (ICDAS II codes 4, 5 and 6) and therefore underestimates the level of caries.

Some authors do not include filled teeth in their DMF scores if they believe them to be associated with atypical caries due to MIH^25^, and therefore obtain lower DMF values, but most atypical restorations in teeth affected by MIH have involved a history of caries even if it cannot be established with certainty at the time of examination. Consequently these authors could be underestimating the DMF, while those who include all fillings in the DMF could be overestimating it. In the present study, following the EAPD criteria^3^, extensive or atypical restorations were included in the MIH diagnosis if they had opacities around the edges or on another surface of the tooth or if MIH criteria applied to another molar.

Diet and oral hygiene play an important role in the etiological factors of caries and the present study observed a rise in mean DMFS as the intake of cariogenic foods increased. The multifactorial analysis found that cariogenic food intake and the presence of severe MIH were significantly related to higher DMFS scores, but that mild MIH showed no association with dental caries.

Preventive measures against dental caries involve improving oral hygiene and changing the child’s dietary habits. Applying fluoride as a varnish or in trays, and in toothpaste or rinses, is also effective. Nevertheless, it is also important to treat active lesions, whether by remineralizing an incipient or chronic lesion or by filling a cavitated lesion. It is very important to monitor teeth with signs of MIH to avoid their premature loss from dental caries, examining primary molars for hypomineralization and, if they are affected, monitoring the development of the permanent first molars^49^. These children should also be included in a high caries risk follow-up protocol.

The present study examined the association between MIH and dental caries and found an association between dental caries and the presence of surfaces affected by severe MIH, which should be considered a risk factor within the multifactorial etiology of caries.
